# A spatiotemporal analysis of acoustic interactions between great reed warblers (*Acrocephalus arundinaceus*) using microphone arrays and robot audition software HARK

**DOI:** 10.1002/ece3.3645

**Published:** 2017-12-10

**Authors:** Reiji Suzuki, Shiho Matsubayashi, Fumiyuki Saito, Tatsuyoshi Murate, Tomohisa Masuda, Koichi Yamamoto, Ryosuke Kojima, Kazuhiro Nakadai, Hiroshi G. Okuno

**Affiliations:** ^1^ Graduate School of Informatics Nagoya University Nagoya Japan; ^2^ Center for Open Innovation Research and Education Graduate School of Engineering Osaka University Suita Japan; ^3^ IDEA Consultants, Inc. Osaka Japan; ^4^ IDEA Consultants, Inc. Fukuoka Japan; ^5^ IDEA Consultants, Inc. Nagoya Japan; ^6^ Department of Biomedical Data Intelligence Graduate School of Medicine Kyoto University Kyoto Japan; ^7^ Honda Research Institute Japan Co., Ltd. Wako Saitama Japan; ^8^ Department of Systems and Control Engineering School of Engineering Tokyo Institute of Technology Meguro‐ku Tokyo Japan; ^9^ Graduate School of Creative Science and Engineering Faculty of Science and Engineering Waseda University Shinjuku‐ku Tokyo Japan

**Keywords:** HARK, microphone array, robot audition, soundscape partitioning, the great reed warbler, transfer entropy

## Abstract

Acoustic interactions are important for understanding intra‐ and interspecific communication in songbird communities from the viewpoint of soundscape ecology. It has been suggested that birds may divide up sound space to increase communication efficiency in such a manner that they tend to avoid overlap with other birds when they sing. We are interested in clarifying the dynamics underlying the process as an example of complex systems based on short‐term behavioral plasticity. However, it is very problematic to manually collect spatiotemporal patterns of acoustic events in natural habitats using data derived from a standard single‐channel recording of several species singing simultaneously. Our purpose here was to investigate fine‐scale spatiotemporal acoustic interactions of the great reed warbler. We surveyed spatial and temporal patterns of several vocalizing color‐banded great reed warblers (*Acrocephalus arundinaceus*) using an open‐source software for robot audition HARK (Honda Research Institute Japan Audition for Robots with Kyoto University) and three new 16‐channel, stand‐alone, and water‐resistant microphone arrays, named DACHO spread out in the bird's habitat. We first show that our system estimated the location of two color‐banded individuals’ song posts with mean error distance of 5.5 ± 4.5 m from the location of observed song posts. We then evaluated the temporal localization accuracy of the songs by comparing the duration of localized songs around the song posts with those annotated by human observers, with an accuracy score of average 0.89 for one bird that stayed at one song post. We further found significant temporal overlap avoidance and an asymmetric relationship between songs of the two singing individuals, using transfer entropy. We believe that our system and analytical approach contribute to a better understanding of fine‐scale acoustic interactions in time and space in bird communities.

## 1 INTRODUCTION

Acoustic interactions are important for understanding communication among species and individuals in songbird communities from the viewpoint of soundscape ecology (Gasc, Francomano, Dunning, & Pijanowski, 2016). In particular, the temporal dynamics of vocalizations are of interest because they are known to have ecological and behavioral implications (Catchpole & Slater, 2008). Birds may divide up sound space in such a manner that they tend to avoid overlap with the songs of other bird species or individuals in order to communicate with neighbors efficiently. There have been empirical studies on the temporal sound space partitioning or overlap avoidance of singing behaviors of song birds across various time scales (Araya‐Salas, Wojczulanis‐Jakubas, Phillips, Mennill, & Wright, 2017; Brumm, 2006; Cody & Brown, 1969; Ficken, Ficken, & Hailman, 1974; Fleischer, Boarman, & Cody, 1985; Masco, Allesina, Mennill, & Pruett‐Jones, 2016; Planqué & Slabbekoorn, 2008; Popp, Ficken, & Reinartz, 1985; Suzuki, Taylor, & Cody, 2012; Yang, Ma, & Slabbekoorn, 2014). We are interested in clarifying the dynamics underlying the process as an example of complex systems based on short‐term behavioral plasticity (Tobias, Planqué, Cram, & Seddon, 2014) from both theoretical (Suzuki & Arita, 2014) and empirical standpoints (Suzuki & Cody, 2015; Suzuki, Hedley, & Cody, 2015). Traditionally, researchers have used a standard single‐channel microphone to record bird songs and manually analyzed the recording to study temporal pattern of the songs. However, it is problematic to manually collect spatiotemporal patterns of acoustic events in natural habitats using data derived from a single‐channel recording of several species singing simultaneously.

Using a microphone array is a promising approach to acoustically monitor wildlife that produce sounds (Blumstein et al., 2011) because it can provide directional or spatial information of vocalizations from recordings. There have been several empirical studies to spatially localize bird songs using multiple microphones for playback experiments (Mennill, Battiston, & Wilson, 2012; Mennill, Burt, Fristrup, & Vehrencamp, 2006) and localization of songs of antbirds in the 2D (Collier, Kirschel, & Taylor, 2010) and 3D spaces (Harlow, Collier, Burkholder, & Taylor, 2013). Araya‐Salas et al. (2017) recently showed that coordinated singing in lekking long‐billed hermits depends on the distance between individuals, using six stereo microphones to roughly estimate the distance between birds. Hedley, Huang, and Yao (2017) also successfully showed a 3D direction‐of‐arrival estimation of up to four simulated birds singing, using two stereo field recorders. Our intent is to further investigate the usability of microphone arrays to study fine‐grained spatiotemporal interactions of bird songs such as for soundscape partitioning.

In this study, we investigated spatiotemporal patterns of the great reed warblers (*Acrocephalus arundinaceus*, GRWA) by combining microphone arrays and an open‐source robot audition system for localization and separation. The singing behavior of this species has been extensively investigated because of the rich variety of song repertoires and its complexity (Forstmeier, Hasselquist, Bensch, & Leisler, 2006; Forstmeyer & Leisler, 2004; Hasselquist, Bensch, & von Schantz, 1996). However, as far as we know, no study has quantitatively discussed the existence of temporal partitioning of the sound space among neighboring individuals of this species.

We are developing an easily available and portable system called HARKBird (Suzuki, Matsubayashi, Hedley, Nakadai, & Okuno, 2016; Suzuki, Matsubayashi, Nakadai, & Okuno, 2017). It automatically extracts bird songs and provides the direction of arrival (DOA) of each localized song, both of which are useful to grasp the soundscape around the microphone array. HARKBird consists of a standard laptop PC with open‐source software for robot audition HARK (Honda Research Institute Japan Audition for Robots with Kyoto University; Nakadai, Okuno, & Mizumoto, 2017; Nakadai et al., 2010) combined with a low‐cost and commercially available microphone array.

Here, we describe the use of HARKBird to localize singing birds in a 2D space in the field. A preliminary analysis of spatial localization with the 3 eight‐channel microphone arrays showed a reasonable accuracy in estimating the location of the song posts of the GRWAs (Matsubayashi et al., 2017). In this study, we use the data obtained from three newly developed 16‐channel, stand‐alone, and water‐resistant microphone arrays, named DACHO, to automatically record bird songs in the field for a detailed analysis on its intraspecific competition. We also improve algorithms for localization. We first report the performance of our system. We then examine the temporal localization performance by comparing the beginning and ending timing of localized song with manually annotated data. Lastly, we examine the temporal overlap avoidance between the focal individuals based on manually annotated data using randomization tests (Araya‐Salas et al., 2017; Masco et al., 2016) and transfer entropy (Schreiber, 2000) for analyzing the information flows in these complex systems (Bossomaier, Barnett, Harré, & Lizier, 2016).

## 2 MATERIALS AND METHODS

### 2.1 Bird observation

**Figure 1:**
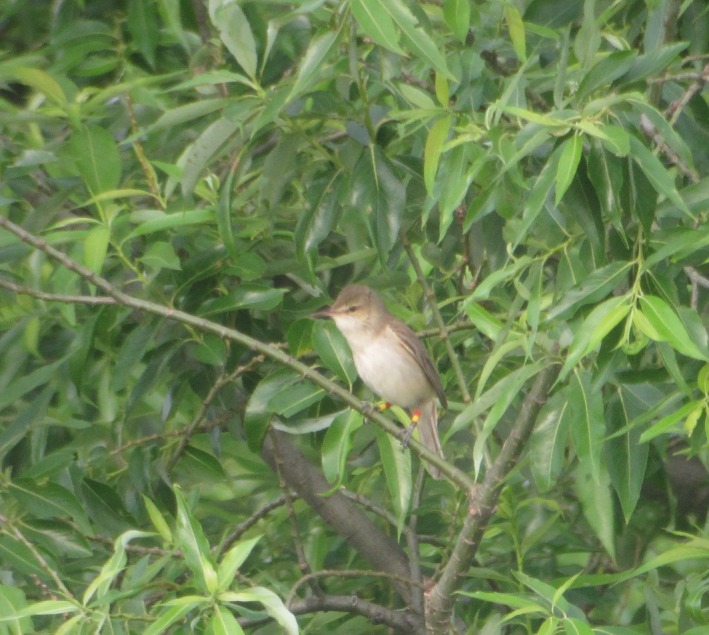
A color‐banded individual of the great reed warbler (RYB)

Males of GRWA (Figure 1) declare and defend their territories with loud and persistent songs during the breeding season. Studies have shown that the repertoires size may play an important role in attracting females and to warn off other potential rivals in the neighborhood (Forstmeier et al., 2006; Forstmeyer & Leisler, 2004; Hasselquist et al., 1996).

**Figure 2:**
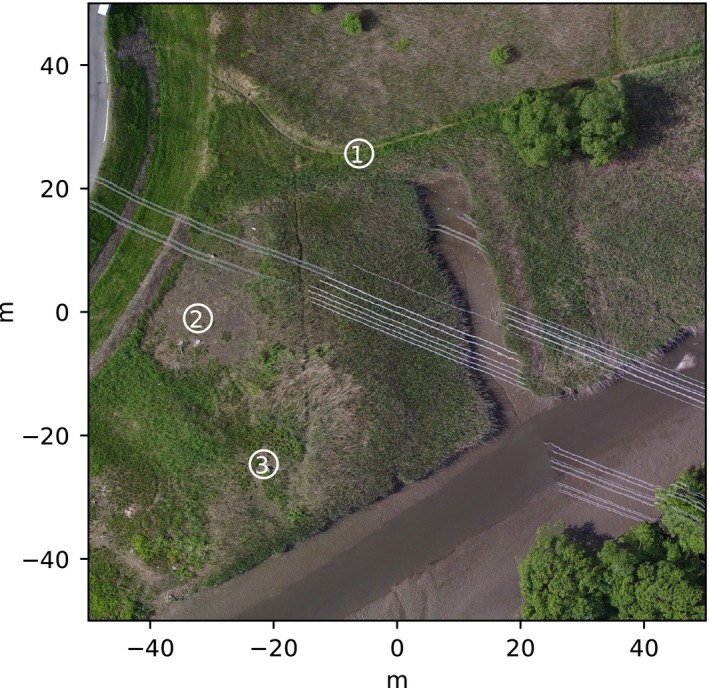
The 2D imagery of the study area. The numbered circles represent the location of the three 16‐channel microphone arrays

We conducted a bird survey in 18 May 2016, during the early breeding season, on a bank of the Ibi river, Kaminogo district, Mie prefecture in central Japan (35°34′59″N, 136°06′29″E). Figure 2 shows a map of the study site. The observations were conducted over approximately 6 hr starting at 6:00 a.m. Each of 17 observation sessions was 20 min long. Using spotting scopes, observers recorded the identification of the bird based on the color bands tagged to both legs, location, activities, and timing of each activity of the bird. Observers determined the location of each bird using landmarks on drone imagery taken by ourselves and marked posts in the field. Positions of landmarks were measured using a GPS (Trimble R10 GNSS; Trimble Inc., California, USA). It is difficult to identify the exact beginning and end of each song, so we instead reported the beginning and the end times of a sequential series of songs including short breaks in‐between songs typically lasting for a few minutes at each observed location.

Three GRWA males within our study area had been captured using a mist net for the purpose of this research and banded for identification prior to this recording experiment, two of which were visually confirmed during recording sessions (Figure 1). We confirmed the presence of five individuals at one point of our experiments, two of which were banded males. According to the field observation, there were a few additional males in the study area, at least one of which was visually confirmed to be a male without bands. This unbanded male briefly flew in and out of the study area, typical behavior of a young male floater in search of vacant territory (Mérő & Žuljević, 2017).

Observed data were classified into four categories: the songs of RYB, RGY, other individuals except for RYB and RGY (OTH), and unknown individuals (UNK). RYB and RGY represent the songs of color‐banded individuals whose bands are red‐yellow‐blue and red‐green‐yellow, respectively. OTH includes individuals of the same species that were not color‐banded. UNK includes individuals of GRWA, but it was not sure whether they are color‐banded or not; thus, it may include RYB and RGY. In cases where multiple individuals were singing simultaneously, we relied on localized results to help distinguish individuals.

### 2.2 Recording with microphone arrays and song post localization

**Figure 3:**
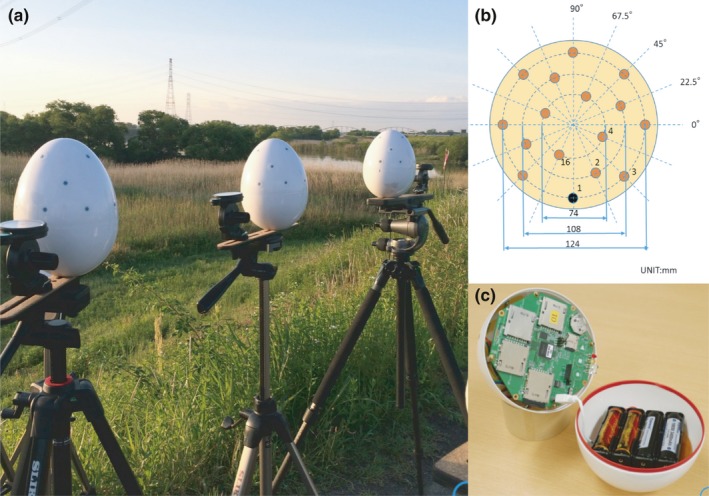
The 16‐channel microphone arrays (a) Each of the three arrays was placed on the top of a tripod on the riverbank. (b) The geometry of the 16‐channel microphones on the frame. (c) The internal structure of the array

We used 16‐channel, stand‐alone, and water‐resistant microphone arrays, named DACHO, specifically developed for bird observations in the field (WILD‐BIRD‐SONG‐RECORDER; SYSTEM IN FRONTIER Inc., Tokyo, Japan). Each array consists of 16 microphones, arranged within an egg‐shaped frame, which is 17 cm in height and 13 cm in width (Figure 3). It records using a 16‐channel, 16 bit, 16 kHz format. Recorded raw data are stored in SD cards and can be exported in either a raw or wave format for further analysis using customized software on a PC to which the array is connected with the USB interface. One can schedule a recording by preparing the time settings in a micro‐SD card. See Table 1 for the specification of the microphone array. We placed three microphone arrays in the reed marsh where the GRWA inhabit (Figure 2) and conducted a scheduled recording for each observation session.

**Table 1:** The specification of the microphone array

Channels	16
Sensitivity	−18 ± 3 dBV/Pa
SNR	63 dB(A)
Sampling rate	16.0 kHz
Resolution	16 bit
Battery	Li‐ion (18650) × 6
Memory	4 SD card slots
Size	170 mm (height) × 130 mm (radius)
Weight	650 g
Power consumption	2 mA (stand by), 400 mW (recording)

**Figure 4:**
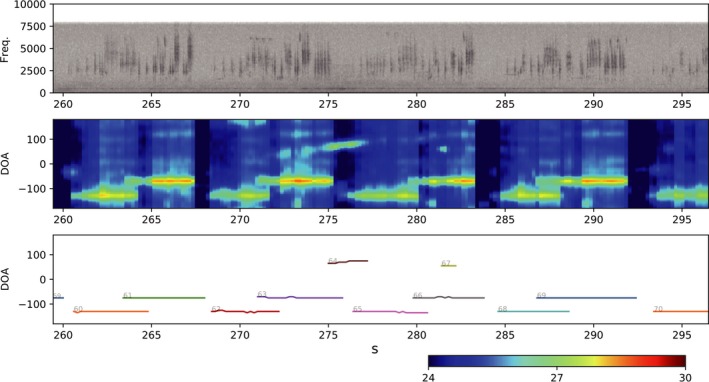
An example of DOA estimation of sound sources by HARKBird. The top panel shows the spectrogram of a channel of the original recording for about 40 s in the session 13 recorded by the second microphone array. The middle panel shows the MUSIC spectrum, which represents the confidence level of the sound existence, calculated for each time and direction. Each line in the bottom panel shows the time and the direction of the localized sources, which is used for the spatial localization

We used HARKBird^1^ to estimate the DOA of the sound sources acquired from each microphone array. The sound source localization algorithm of HARK is based on the MUltiple SIgnal Classification (MUSIC) method (Schmidt, 1986) using multiple spectrograms with the short‐time Fourier transformation. See Suzuki et al. (2017) for additional details of HARKBird and Nakadai et al. (2017, 2010) for HARK. We adjusted the parameters to localize songs of the GRWAs as much as possible while suppressing other sounds (e.g., noise and songs of other bird species).^2^ An example of DOA of sound sources estimated by HARKBird (Figure 4) shows that the two GRWA individuals were singing alternately at directions different from a microphone array.

**Figure 5:**
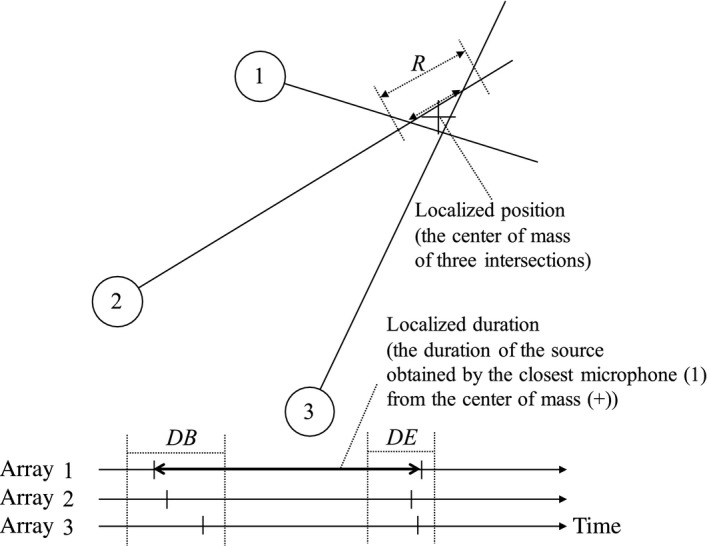
Spatiotemporal localization of sound sources

We used an improved algorithm for spatial localization based on the one adopted in Matsubayashi et al. (2017) (Figure 5). At each time frame DT (=0.2 s) of the localization process, we assumed that a half line arose from each microphone array toward the DOA estimated sound source. We used the center of mass of the three intersections of those three half lines as the estimated location of a 2D localized sound indicated by a “+” in Figure 5.

Because multiple sound sources can be localized at each time frame, we have to exclude cases when microphone arrays estimated DOA of different sound sources. For this purpose, we adopted the 2D localization result only when it met two requirements. First, the distances between all three intersections were equal or smaller than *R* (=15 m) (indicated as a dotted line). Second, the begin and end timing of all sound sources should be within the range of DB (=6.0 s) and DE (=1.0 s), respectively. The reason that we used a large DB and a small DE is that a song of the GRWA begins with several short introductory notes that may be difficult to localize, while it ends with loud notes that are easily localized. Thus, the end timing is a good signal for discriminating different sound sources.

Finally, we define the song duration of the spatially localized sound as that of the closest microphone array to the center of mass, where the sound was most clearly recorded. For example, in Figure 5, the song duration localized by the microphone array 1 was used. Note that because HARK with our setting requires a silence of 0.8 s to detect the end of each localized sound, we reduced the estimated duration of songs by 0.6 s.

**Figure 6:**
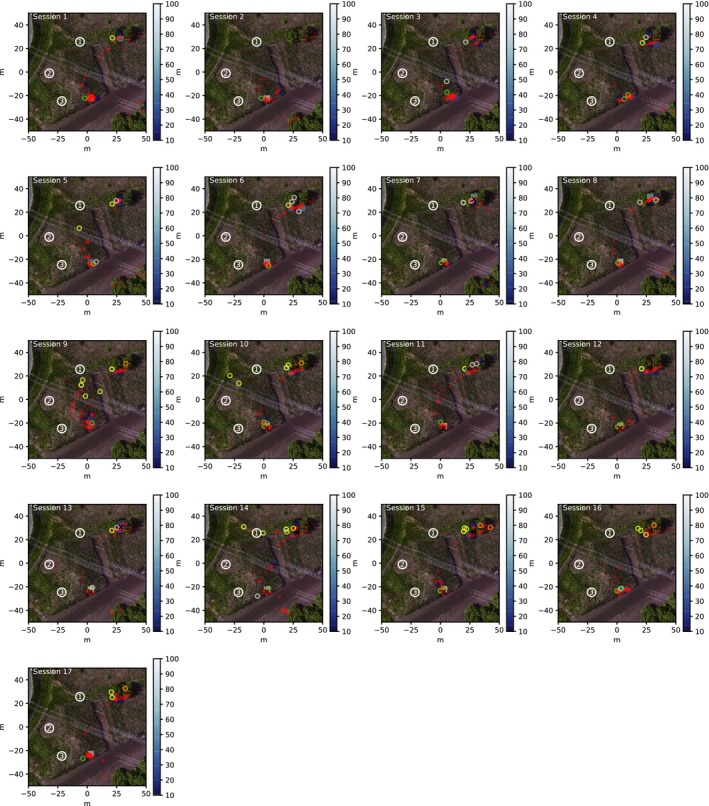
The spatial distribution of localized locations and observed locations in the all 17 recording sessions. A red “+” represents the former (i.e., localized) and colored “o” represents the latter (i.e., observed). Four categories of the observed birds, that is, RYB, RGY, OTH, and UNK are color‐coded by yellow, green, orange, and gray, respectively. Blue rectangles represent the 5 × 5 m^2^ area in which more than 10 sound sources were spatially localized. The brighter color corresponds to the larger number of localized sounds within the area

**Figure 7:**
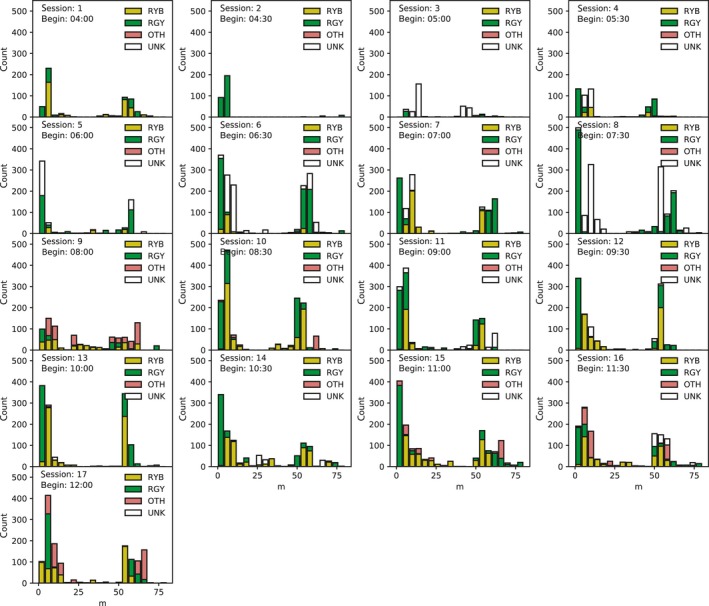
The distribution of observed‐localized distance of songs in all 17 recording sessions. At every time interval of 1 s, we measured the distance between each observed location and the localized location that was the closest to the observed location and visualized a histogram of this distance for each observed individual

From these results, we visualized spatial distributions of the location of the localized sound sources and observed birds in all the 17 sessions (Figure 6). We also calculated the distribution of these observed‐localized distances (Figure 7). Specifically, at every time interval of 1 s, we measured the distance between each observed location and the localized location that was the closest to the observed location and visualized a histogram of this distance for each observed individual.

### 2.3 Temporal analysis

**Figure 8:**
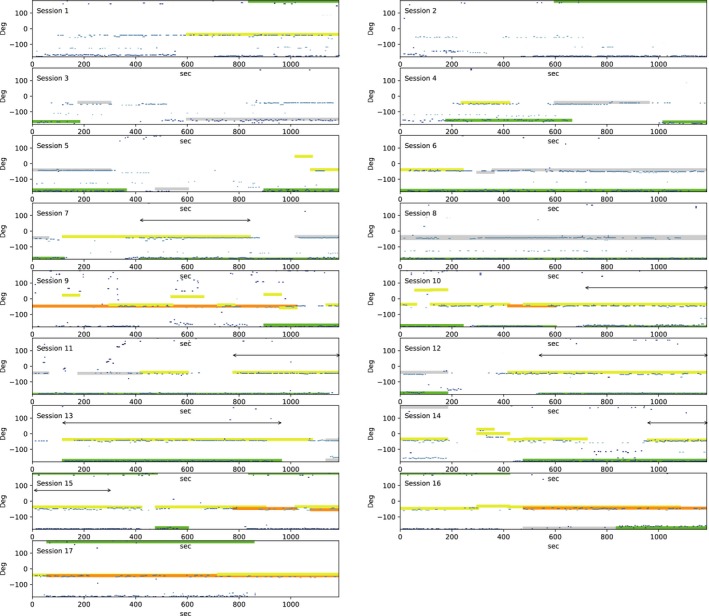
The temporal and directional distribution of localized songs and observed locations in the all 17 recording sessions. The horizontal axis represents time, and the vertical axis represents the direction from the center of mass of the three microphone arrays. The observed durations of RYB, RGY, OTH, and UNK are indicated by thick yellow, green, orange, and gray lines, respectively. Localized sound sources are shown in blue lines, whose brightness corresponds to the distance from the center of mass of the three arrays. Double‐headed arrows represent durations of bouts during which both RYB and RGY were observed and singing actively around their song posts in seven different sessions

To determine whether the temporal soundscape partitioning occurred between RYB and RGY, we compared the durations of bouts during which both individuals were observed and singing actively around their song posts in seven different sessions (Figure 8). We adopted these durations to extract more direct and clearer interactions between these target individuals with minimum interruption of other individuals singing in the neighborhood.

To extract the temporal dynamics of the singing behavior of these two individuals, we presumed that sound sources localized around each bird's song post belong to that bird. Specifically, we used the sources within the range of 15 m (RYB) and 10 m (RGY) from the locations indicated with “x” in Figure 6, respectively. We adopted the larger range for RYB because this individual tended to move more frequently around his song post, whereas the other one remained at one spot. We assigned these sources in a timeline of each individual assuming that there was no time overlap among sound sources. In case of multiple overlapping song intervals, we adopted the one that began earlier. Trained researchers visually and auditorily examined the localization performance using Praat (Boersma, 2001). In this annotation process, we manually adjusted the beginning and end of a song, added mislocalized sound, and removed sounds that were not the songs of the targets.

Using these annotated data, we calculated the localization performance by comparing the localized song durations and the manually annotated song durations. For this analysis, we classified the durations into four categories: true positive (TP) when localized songs were actually observed, false positive (FP) when songs were localized but not observed, true negative (TN) when songs were not localized and not observed, and false negative (FN) when songs were not localized but actually observed. We evaluated the accuracy ((TP+TN)/(TP+FP+TN+FN)) of the results and ROC curves (defined by true‐positive rate (TP/(TP+FN)) and false‐positive rate (FP/(FP+TN)) of individuals.

To examine whether a significant temporal overlap avoidance existed among these individuals, we first focused on the solo singing duration during which only one individual sang, using the Monte Carlo randomization test. We defined *X*_rand_ (*X* ∈ {RYB, RGY}) as the randomized time series of the individual *X* during which the duration of nonsinging intervals were randomly shuffled from the original series. We created 10,000 randomized data of RYB_rand_ and RGY_rand_ and calculated the solo durations for all data. We regarded the proportion of these solo durations that was larger than the observed duration as the *p*‐value for the null hypothesis (no significant overlapping and avoiding overlap). There exist two possibilities in which these individuals actively avoid overlap or actively overlap with each other. We regarded that the former case is significant when the *p*‐value is smaller 0.025 and the latter case is significant when the *p*‐value is larger than 0.975 (two‐tailed test). However, we expect that they were singing alternately (and thus avoiding overlaps) according to human observations.

We also used SONG (Song Overlap Null model Generator), a package of R (Masco et al., 2016), to examine whether there was a significant asymmetric effect from one individual to the other. Using SONG, we classified the overlapped duration of the two individuals (*X* and *Y*) ‘s songs into two: the duration during which a target individual *X* began to sing while the other reference individual *Y* was singing (i.e., *X* actively overlapped with *Y*) and vice versa. We created 10,000 randomized data using the time series *X*_rand_ and *Y*, and calculated the durations during which the randomized target individual *X*_rand_ actively overlapped with *Y* for all data. We defined the proportion of those durations that was smaller than the observed value as the *p*‐value for the null hypothesis. That is, the target individual significantly avoided overlap when the *p*‐value is smaller 0.025 and the target individual actively overlapped when the *p*‐value is larger than 0.975 (two‐tailed test).^3^

To grasp a general trend of overlap avoidance across all the sessions, we calculated a bootstrap estimate of expected–observed duration of overlap for each individual, using the data from all seven sessions.

We further measured the information flow from one individual's singing behavior to another using transfer entropy (Schreiber, 2000), which has been recently used to analyze information flows in complex systems (Bossomaier et al., 2016). Specifically, this measure quantifies the expected amount of directional information flow from one time series to another; the transfer entropy *T*_*Y*→*X*_(*k*,* l*) from a discrete time series *Y*_*t *_= {*y*_*t*_}_*t*=1,2,…_ to another discrete time series *X*_*t*_ = {*x*_*t*_}_*t*=1,2,…_. Given the past *k* values of *X*_*t*_, the amount of reduction in the uncertainty about the future value of *X*_*t*_ (i.e., the reduced entropy of the transition probability of *X*_*t*_) by knowing the past *l* values of *Y*_*t*_ is calculated as follows:

(1) $${\text{TE}}_{Y\to X}\left(k,l\right)\;=\sum_{x_{t}^{k},x_{t+1},y_{t}^{l}}\text{log}\frac{p(x_{t+1}|x_{t}^{k},y_{t}^{l})}{p(x_{t+1}|x_{t}^{k})},$$

where $x_{t}^{k}$ and $y_{t}^{l}$ denote {*x*_*t*−*k*+1_, …, *x*_*t*_} and {*y*_*t*−*l*+1_, …, *y*_*t*_}, respectively. In our case, *X* and *Y* correspond to the time series of singing behavior of individuals *X* and *Y* when we calculated the information flow from the source individual *Y* to the sink individual *X*. To discretize each time series, we created a binary time series by assigning a binary value (1: singing or 0: not singing) to each 0.5‐s time interval.^4^

For a statistical test, we created 10,000 randomized data using the time series *X* and *Y*_rand_ and calculated ${\text{TE}}_{Y_{\mathrm{rand}}\to X}\left(k,l\right)$ for all data. The *p*‐value for the null hypothesis (i.e., the information flow from *Y* to *X* is not significantly larger than randomized ones) refers to the proportion of calculated values that is larger than the observed value TE_*Y*→*X*_ (*k*,* l*).

We expect that if an individual *X* actively avoids overlapping with *Y* (in the sense above), the singing behavior of *X* is expected to depend on the behavior of *Y*, and thus, there should be a significant information flow from *Y* to *X*. In other words, the reference and target individuals correspond to the source and sink individuals, respectively. Because transfer entropy is itself asymmetric measure and it is not natural to assume that the information flow can become significantly smaller than that in the cases with randomized sources, we adopted one‐tailed test here. We also calculated a bootstrap estimate of observed value (TE)–expected (TE_rand_) value of transfer entropy for each individual, using the data from all the seven sessions.

In addition, while we could only observe a single duration (100–800 s) in the session 17 in which RYB, RGY, and OTH were visually observed and actively singing, we further conducted the same analysis on possible pairs of these three individuals to see interactions among them.

## 3 RESULTS

### 3.1 Spatiotemporal distribution

The location of observed birds shows that two color‐banded individuals, RYB and RGY, tended to stay and sang at around their own song posts (Figure 6). RYB sang in a tree and RGY sang within the reed bushes at the waterfront. UNK and OTH were sometimes observed around RYB, implying that these individuals might have competed with RYB as potential rivals.

Areas in which sound sources (represented as “+”) were frequently localized are located in close proximity to each bird's song post reported by human observers, showing that the songs of these individuals were successfully localized. We also see several additional sound sources localized within the study area. Those distant from song posts are expected to be vocalizations of other species or environmental noises. Sounds localized in between the song posts are likely to be caused by a triangulation when the DOAs of different sound sources were used.

There are bimodal peaks in the distribution of this observed‐localized distance in all 17 sessions (Figure 7). The peak around 4–12 m corresponds to the distribution of the distance between the observed bird and the localized location of his song. The average distances between the localized locations and the observed locations which were <30 m were RYB: 8.334 ± 4.814, RGY: 3.514 ± 2.904, OTH: 10.31 ± 4.921, UNK: 9.431 ± 5.098 and (RYB and RGY): 5.457 ± 4.468. The distance of RYB, OTH, and UNK tended to distribute more broadly than that of RGY. This implies that they tended to move around the song post more frequently than RGY, but human observers cannot easily record such a small movement.

The second peak around 50 m corresponds to the cases where there were no sound sources around the song posts. However, this does not necessarily mean that the localization was unsuccessful, because the observed song duration includes not only multiple song durations but also includes short breaks in‐between songs (explained below). We expect that the most of the sources localized from about 50 m away from one song post are expected to be the localized songs at the other song post.

In the temporal and directional distribution of localized sound sources and human observations in all 17 sessions (Figure 8), most of the localized sound sources (shown in blue lines) especially for RYB and RGY aligned well with human observations (indicated by thick lines). It should be noted that the localization results are more fine‐grained than human observations in the sense that they included the begin and end timing of each song. Results also show that RGY tended to stay at a fixed location while singing through all the sessions without any conspecific rival around his song post. At the same time, RYB tended to move frequently around his song post with potential rivals such as the one coded as OTH and UNK. Note that UNK could actually be RYB that went out of the observer's sight for a moment. RYB and OTH were expected to be competing for their song posts.

### 3.2 Temporal localization

To evaluate the accuracy of temporal dynamics of automatically extracted song durations, we examined the durations of bouts during which both individuals were observed to be singing actively around their song posts in seven different sessions. These durations are indicated by double‐headed arrows in Figure 8. In these durations, we visually confirmed the presence of the two singing individuals, RYB and RGY. However, OTH was not visually confirmed and his vocalization in the recordings was considerably rarer than those of RYB and RGY. We believe that these conditions allow us to infer direct competition between the focal individuals. As for the effects of vocalizations of other species on the great reed warbler, we believe that these species dominated the acoustic space around them because of much higher rate of their vocalizations and their loudness.

**Figure 9:**
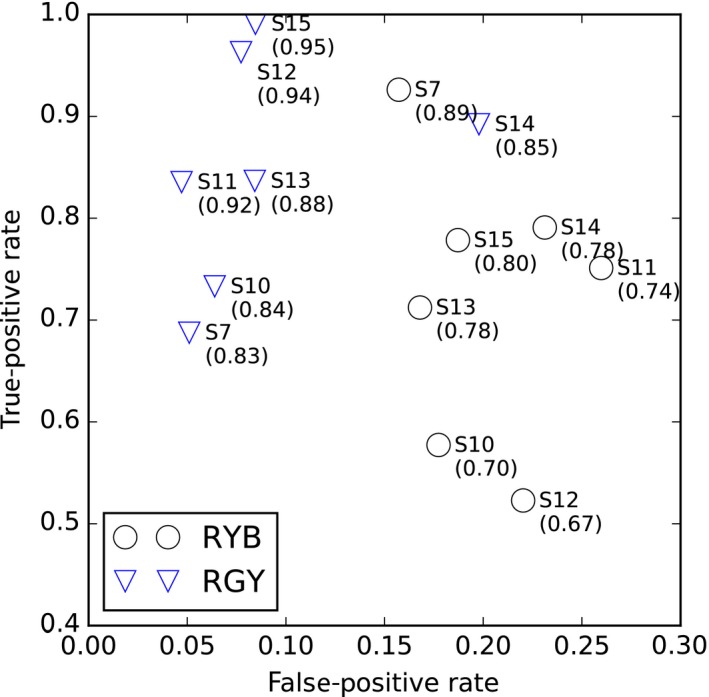
A comparison between annotated and localized song durations. We classified the durations into four categories: true positive (TP) when localized songs were actually observed, false positive (FP) when songs were localized but not observed, true negative (TN) when songs were not localized and not observed, and false negative (FN) when songs were not localized but actually observed. We evaluated the accuracy ((TP+TN)/(TP+FP+TN+FN)) of the results and ROC curves (defined by true‐positive rate (TP/(TP+FN)) and false‐positive rate (FP/(FP+TN)) of individuals. The number next to each point represents the session ID of each duration, and a value with parenthesis represents accuracy of localization result in the corresponding duration

The comparison between annotated and localized song bouts showed that there was a large difference in the accuracy, true‐positive and false‐positive rate of the song extraction between the two (Figure 9). The songs of RGY, the one that sang at one song post, were successfully localized, indicated by high true‐positive rate and low false‐positive rate, which resulted in high accuracy in the range of 0.83 and 0.95 in all seven sessions. High accuracy supports that we can rely on the automatically extracted data for further analyses for this individual, although they still need a minor manual correction (e.g., adjustment of the beginning and end of a song, addition of mislocalized sounds, removal of sounds that were not the songs of the targets).

In contrast, the accuracy of the extracted songs of RYB was in a wider range of 0.67 and 0.89, which is lower than those of RGY. This is due to the fact that true‐positive rate of his songs was lower than that of RGY. Lower true‐positive rate was attributed to the behavior of RYB. This individual frequently flew around his territory or went behind the tree where his song post was located, which made the localization difficult and increased FN. The lower accuracy is also due to higher false‐positive rate. This could be caused accidentally when other species (e.g., Japanese bush warbler (*Horornis diphone*), Coal Tit *Periparus ater insularis*) or other males of the GRWA occasionally sang in a similar direction toward the microphone 1 because songs of RYB and these individuals could be localized as a single source. Despite these limitations, sufficient accuracy values indicate that they can serve as initial estimates for successive manual annotation.

### 3.3 Temporal overlap avoidance

Using annotated data, we investigated the temporal interactions between RYB and RGY based on their annotated song timings (Table 2). These two birds avoided overlapping indicated by their significantly longer solo singing duration than expected by chance in almost all of the seven recording sessions (7, 12, 13, 15; *p* < .001, 10, 11; *p *< .01). This soundscape partitioning might be realized by the asymmetric tendency of the overlap avoidance behavior. Table 3 shows that RGY actively avoided to begin singing while RYB was singing in the two recording sessions (7; *p* < .001, 13; *p* < .01). In contrast, RYB actively avoided RGY in only one recording session (15; *p *< .01). A bootstrap estimate of expected duration–observed duration of overlap for each individual showed that the values were significantly positive (mean = 7.471, *p* = .005, 95% CI [1.780, 12.06] (percentile confidence interval based on 10,000 resampling) (RYB); mean = 17.24, *p* = .000, 95% CI [9.703, 26.13] (RGY)), meaning that both RYB and RGY actively avoided an overlap. We further calculated a bootstrap estimate of the difference in the observed/expected ratio of overlapped duration between RYB and RGY over all the seven sessions. The difference was significantly positive (mean = 0.2272, *p* = .0086, 95% CI [0.0490, 0.4098]), showing that RGY more actively avoided overlap more frequently than RYB did. Overall, we conclude that both RYB and RGY were actively avoiding an overlap. Of the two, RGY was more active than RYB in avoiding the opponent.

**Table 2:** Temporal soundscape partitioning

Session	Duration (s)	Vacant (s)	Overlapped (s)	Solo (s)	Solo_rnd (s)	*p*‐value
7	420	81.8	67.1	271.1	209.5	.0000**
10	466	102.1	88.1	275.7	230.5	.0075*
11	406	148.4	36.6	221.0	188.3	.0014*
12	646	186.7	85.0	374.3	311.9	.0002**
13	840	295.1	78.3	466.7	390.7	.0001**
14	226	55.9	41.7	128.5	108.5	.0460
15	290	83.8	21.8	184.5	138.4	.0000**

Single asterisk (*) denotes significance at p < 0.01, and double asterisk (**) denotes significance at p < 0.001.

**Table 3:** The asymmetric and active overlap avoidance

Session	Reference	Target	Observed (s)	Expected (s)	*p*‐value	Expected–observed	Obs./exp. (RYB)‐obs./exp. (RGY)
7	RYB	RGY	18.77	55.40	.0000**	mean = 17.2495% CI = [9.703, 26.13]*p* = .0000**	mean = 0.227295% CI = [0.0490, 0.4098]*p* = .0086*
10			46.66	57.31	.1605		
11			14.29	25.31	.0328		
12			39.70	56.94	.0293		
13			26.03	57.08	.0015*		
14			14.42	21.76	.1395		
15			9.71	16.70	.0822		
7	RGY	RYB	48.34	41.67	.7829	mean = 7.47195% CI = [1.780, 12.06]*p* = .0051*	
10			41.45	53.85	.1063		
11			22.34	28.57	.1213		
12			45.29	57.95	.0705		
13			52.23	64.31	.0980		
14			27.24	28.74	.4115		
15			12.05	26.21	.0041*		

“Expected‐observed” represents a bootstrap estimate of expected duration‐observed duration of overlap for each individual, using the data from all the seven sessions. “obs./exp. (RYB)‐Obs./exp. (RGY)” represents a bootstrap estimate of the difference in the observed/expected ratio of overlapped duration between RYB and RGY. Single asterisk (*) denotes significance at p < 0.01, and double asterisk (**) denotes significance at p < 0.001.

The information flow between the two individuals measured using transfer entropy (Table 4) shows that there was a significant information flow from RYB to RGY in the four recording sessions (7, 11, 12, 14; *p *< .05); thus, the future singing behavior of RGY could be predicted by the behavior of RYB. The opposite was not true for the other case. A bootstrap estimate of observed value ‐ expected value of transfer entropy for each individual showed that the value of RYB→RGY was significantly positive while that of RGY→RYB was insignificant (mean = 0.006083, *p* = .000, 95% CI [0.003257, 0.009243] (RYB→RGY); mean = −0.0003615, *p* = .6303, 95% CI [−0.002543, 0.0018] (RGY→RYB)), meaning that there was a significant information flow from RYB to RGY.

**Table 4:** The transfer entropy from an individual to the other

Session	Source	Sink	TE	TE_rnd	*p*‐value	TE‐TE_rnd
7	RYB	RGY	0.0145	0.0034	.0258*	mean = 0.00608395% CI = [0.003257, 0.009243]*p* = .0000*
10			0.0055	0.0032	.1761	
11			0.0071	0.0018	.0255*	
12			0.0089	0.0014	.0040*	
13			0.0032	0.0010	.0559	
14			0.0169	0.0045	.0375*	
15			0.0048	0.0030	.2033	
7	RGY	RYB	0.0043	0.0030	.2244	mean = −0.000361595% CI = [−0.002543, 0.0018]*p* = .6303
10			0.0007	0.0028	.7012	
11			0.0006	0.0024	.7602	
12			0.0029	0.0015	.1586	
13			0.0011	0.0014	.4126	
14			0.0009	0.0064	.8381	
15			0.0075	0.0029	.0816	

TE represents transfer entropy from the source individual to the sink individual, and TE_rnd represents the corresponding entropy when the temporal dynamics of the source individual was randomized. “TE‐TE_rnd” represents a bootstrap estimate of observed value‐expected value of transfer entropy for each individual, using the data from all the seven sessions. Single asterisk (*) denotes significance at p < 0.05.

It should be noted that both active overlap avoidance and the information flow demonstrated a similar trend. While the information transfer itself does not tell us about its mechanism, the information flow from RYB to RGY is expected to be associated with the active overlap avoidance behavior of RGY, which depends on the preceding song of RYB.

In addition, we further conducted the same analysis on possible pairs of RYB, RGY, and OTH in a single duration (100–800 s) in the session 17 in which these individuals were visually observed and actively singing. Results were summarized as follows: There was significant overlap avoidance between all pairs (*p* < .025). However, RYB was neither actively overlapping nor avoid overlapping with the other two individuals while both RGY and OTH were actively avoiding overlap with all the others (*p *< .025). There was significant information flow from RYB to OTH, OTH to RYB, and OTH to RGY (*p* < .025).

These results show that RYB were not actively avoiding overlapping with the others, while RGY and OTH were actively avoiding overlap with RYB. This supports our claim that there exists asymmetric relationship among them. In other words, RYB was a driver individual in this soundscape of GRWA songs.

## 4 DISCUSSION

We examined spatiotemporal relationship between two GRWAs in a natural habitat using three 16‐channel microphone arrays and open‐source sound source localization software developed for robot audition, HARK. Our system successfully localized many songs of GRWA that sang at two different song posts within the study area. Despite a relatively large distance among microphone arrays, each of which was placed 30–70 m apart from each other (Figure 6), most songs of focal individuals (RYB and RGY) were localized within a range of 5.5 ± 4.5 m away from the location of observed song posts.

Mennill et al. (2012) constructed an array of multiple commercial stereo recorders and synchronized recorded sounds derived from four Song Meters (SM2‐GPS; Wildlife Acoustics Inc., Concord, MA, USA) to generate eight‐channel data. After manually extracting wildlife vocalizations, they estimated the spatial location of each sound source using the cross‐correlation method. Their experiments using play back calls showed that the locational accuracy was much higher when the sound source was inside the area enclosed by recorders, as compared to outside the boundary. While the localization accuracy highly depends on ecological situations and properties of localized songs, the locational accuracy of 10.22 ± 1.64 m for sounds broadcasted outside the arrays using their system is comparable to our localization accuracy. Collier et al. (2010) achieved much higher localization accuracy when they used the 8 four‐channel microphone arrays and simple sounds within the perimeter of the area. We expect that we can also obtain much higher localization accuracy by either increasing the number of microphone arrays or enclosing focal individuals within the area surrounded by the microphone arrays.

Acoustic monitoring using automatic recording devices has been of interest, but practical comparisons of manual and autonomous methods for bird vocalizations are still limited (Digby, Towsey, Bell, & Teal, 2013). We extracted the temporal pattern of song bouts in which two color‐banded individuals were actively singing at their song posts using the localized results. The extracted pattern included the begin and end time of each song, which cannot be easily obtained from human observations. In our experiments, localization accuracy was average 0.89 for one bird that stayed at one song post, and it decreased to 0.77 for the other one that frequently moved around his territory.

As far as we know, no study has analyzed the fine‐grained temporal dynamics of multiple singing birds using these localization results. We expect that we can improve the localization accuracy under challenging conditions; that is, multiple individuals are singing simultaneously in the similar direction, by improving localization algorithms. Localization accuracy reflected a large difference in singing behavior of the two GRWAs: the lower accuracy for RYB and the higher accuracy for RGY.

The difference in their behaviors might be affected by their relationships with neighbors. According to the human observations, there were no other individuals around the song post of RGY and there were other individuals (OTH and UNK) close to the song post of RYB. Thus, there might have existed a strong competitive relationship between RYB and the neighbors, which forced RYB to move around more frequently than RGY. The lower extraction accuracy of RYB might have attributed to such constant movements of RYB, because the human observers cannot record each of the minor positional changes. Our system might be able to extract such detailed spatial information of individuals.

The temporal interactions among individuals, such as temporal overlap avoidance among neighboring birds (Araya‐Salas & Smith‐Vidaurre, 2017; Araya‐Salas et al., 2017; Brumm, 2006; Cody & Brown, 1969; Ficken et al., 1974; Masco et al., 2016; Planqué & Slabbekoorn, 2008; Popp et al., 1985; Suzuki et al., 2012; Yang et al., 2014), have long been a focus of interest in ornithology. Analysis shows that the two GRWAs were singing alternately in our study area. We also found that such coordinated singing process was realized by asymmetric effects among them. The randomization tests and a bootstrap estimate of the duration of the active overlap showed that RGY more actively avoided overlapping with RYB, while the RYB seemed less affected by RGY. The analysis of temporal dynamics of RYB, RGY, and OTH further showed that RYB were not actively avoiding overlap with all the others while RGY and OTH were actively avoiding overlap with RYB. This supports our claim that there exists asymmetric relationship among them. In other words, RYB was a driver individual in this soundscape of GRWA songs.

Measuring coordination behaviors among birds has been complicated, because distinguishing vocal coordination from patterns arising by chance is challenging using conventional statistical approaches (Araya‐Salas et al., 2017; Masco et al., 2016). Recently, the concept of transfer entropy has been used to analyze asymmetric relationships among components of various complex systems (e.g., cellular automata, small‐world networks, and swarms; Bossomaier et al., 2016). In our study, the statistical analysis of information transfer from one individual to another showed a similar tendency of active overlap avoidance. A stronger information flow from RYB to RGY indicates that the future behavior of RGY can be predicted more accurately by knowing the behavior of RYB. We could extract these song‐by‐song interactions using the short time interval (second) to discrete continuous time data. Our results imply that transfer entropy is useful for measuring such short‐term interactions based on the behavioral plasticity in bird vocalizations.

Interaction networks of birds are also a topic of interest (Stowell, Gill, & Clayton, 2016; Tobias et al., 2014). Although we mainly focused on the songs of two focal individuals, it is plausible that we should investigate the songs of other males in the study area for a full assessment of the complex system composed of multiple birds. An additional analysis on three individuals RYB, RGY, and OTH supports our claim that there exists a directional network of interactions among them. In another study conducted in a forest in Japan, we found a statistically significant overlap avoidance in three bird species and a symmetric effect from one species to another (Suzuki et al., 2017). We believe that large‐scale experiments using our system enables us to extract spatiotemporal behaviors of a greater number of individuals to obtain a network of the information flow as well as spatial relationships among them. Actually, we conducted large‐scaled experiments using a greater number of microphone arrays in the field. According to preliminary analysis, one of the problems of the current 2D localization method based on triangulation is a combinatorial explosion of possible locations of sound sources due to the increased number of DOAs recognized by many arrays. It is our future work to resolve this problem.

We could successfully localize songs of the great reed warbler for mainly two technical reasons, both of which were critical to avoid localizing unnecessary sound sources including songs of other species. First, we could limit the minimum frequency range relatively high. Second, we could use the threshold values for MUSIC spectrum relatively large. Thus, conducting sound source localization with different species‐specific settings would enable us to obtain vocalizations of other species effectively. The effectiveness of tuning localization parameters was assessed in our test we conducted for forest birds (Suzuki et al., 2016). We found that different species were successfully localized depending on the settings of parameters.

In this study, we manually identified songs of neighboring individuals (i.e., RYB, OTH, and UNK) using both automatic localization results and human observation. Automated sound recognition is a recent development in bioacoustics and bird monitoring (Jahn, Ganchev, Marques, & Schuchmann, 2017) and a benchmark problem in machine learning (Goëau, H., Vellinga, Planqué, & Joly, 2016). We expect that song classification based on localized and separated sound sources (Kojima, Sugiyama, Suzuki, Nakadai, & Taylor, 2016) will contribute to track the behaviors of conspecific individuals in our system. Integrating these techniques into our automatic localization system will enable us to investigate fine‐grained acoustic interactions in time and space in bird communities, for a deeper understanding of soundscape ecology (Gasc et al., 2016).

## ETHICS STATEMENT

The protocols followed for the survey and to capture individuals of the great reed warbler were approved by the Division of Wildlife, the Chubu Regional Environment Office, the Ministry of the Environment, Japan (Permit Number 1604141).

## DATA ACCESSIBILITY

Data available from the Dryad Digital Repository: https://doi.org/10.5061/dryad.n378d

## CONFLICT OF INTEREST

The authors declare no competing financial interests.

## AUTHOR CONTRIBUTIONS

R.S., R.K., and S.M. conducted field recording using 16‐channel microphone arrays. T.Masuda captured and color‐banded individuals of the great reed warbler. F.S., T.Masuda, K.Y., and S.M. conducted bird observations. T.Murate advised on the design of field experiments. K.N. and H.G.O. developed microphone arrays and advised on the development of the experimental system including HARKBird. R.S. analyzed the results and wrote the manuscript with support from all authors.

## ENDNOTES

1 http://www.alife.cs.is.nagoya‐u.ac.jp/~reiji/HARKBird/

2 We used (1) the expected number of sound sources for the MUSIC method: three sources, (2) the lower bound frequency for the MUSIC method: 2,200 Hz, and (3) the threshold for source tracking: 26.5. See (Suzuki et al., 2017) or the website of HARKBird for the details of each parameter.

3 We used the SONG package for this process, but adopted a different definition of the *p*‐value from the one adopted in (Masco et al., 2016) for our primary purpose, as described in the main text.

4 We used *k *= *l *=* *1.
